# Comprehensive Analysis of the Catalase (*CAT*) Gene Family and Expression Patterns in Rubber Tree (*Hevea brasiliensis*) under Various Abiotic Stresses and Multiple Hormone Treatments

**DOI:** 10.3390/ijms25010070

**Published:** 2023-12-20

**Authors:** Wencai Yu, Guanghong Kong, Huajin Ya, Ligang He, Yu Wu, Hanyao Zhang

**Affiliations:** 1 Yunnan Key Laboratory of Sustainable Utilization Research on Rubber Tree, National and Local Joint Engineering Research Center of Breeding and Cultivation Technology of Rubber Tree, Yunnan Institute of Tropical Crops, Jinghong 666100, China; ywckgh@163.com (W.Y.); kgh.003@163.com (G.K.); yahuajin@swfu.edu.cn (H.Y.); ynhlg@163.com (L.H.); 2 Key Laboratory of Conservation and Utilization of Southwest Mountain Forest Resources, Ministry of Education, Southwest Forestry University, Kunming 650224, China

**Keywords:** catalase (CAT), gene family, abiotic stress, exogenous hormone, abscisic acid (ABA), methyl jasmonate (MeJA)

## Abstract

Catalase (CAT) is one of the key enzymes involved in antioxidant defense systems and mainly scavenges H_2_O_2_ and plays a vital role in plant growth, development, and various adverse stresses. To date, a systematic study of the *CAT* gene family in rubber tree has not been reported. In this study, five *HbCAT* gene family members were identified from the rubber tree genome, and these were mainly clustered into two subfamilies. Gene structure and motif analysis showed that exon-intron and motif patterns were conserved across different plant species. Sequence analysis revealed that *HbCAT* proteins contain one active catalytic site, one heme-ligand signature sequence, three conserved amino acid residues (His, Tyr, and Asn), and one peroxisome-targeting signal 1 (PTS1) sequence. Fragment duplication is a selection pressure for the evolution of the *HbCAT* family based on Ka/Ks values. Analysis of cis-acting elements in the promoters indicated that *HbCAT* gene expression might be regulated by abscisic acid (ABA), salicylic acid (SA), and MYB transcription factors; furthermore, these genes might be involved in plant growth, development, and abiotic stress responses. A tissue-specific expression analysis showed that *HbCAT*s gradually increased with leaf development and were highly expressed in mature leaves. Gene expression profiling exhibited the differential expression of the *HbCATs* under cold, heat, drought, and NaCl stresses. Our results provide comprehensive information about the *HbCAT* gene family, laying the foundation for further research on its function in rubber tree.

## 1. Introduction

In nature, plants face severe threats from various environmental stresses, including biotic and abiotic stresses [1]. Environmental stresses alter the cellular redox stationary phase, leading to the production of large amounts of reactive oxygen species (ROS), such as superoxide anions (O^2−^), hydroxyl radicals (OH^−^), per-hydroxyl radicals (HO^2−^), alkoxyl radicals, hydrogen peroxide (H_2_O_2_), singlet oxygen (^1^O_2_), and other oxygen radicals [2,3,4]. ROS plays an indispensable role as signaling molecules in a majority of biological processes, such as the regulation of plant growth, development, and response to biotic and abiotic stresses [2,3,5]. However, the excessive production of ROS in plant cells leads to lipid peroxidation, as well as damages nucleic acid and protein structures, and impedes carbohydrate synthesis and metabolism, which, in turn, affects plant growth and development, ultimately leading to lower yields [6,7,8].

To adapt to adverse environmental conditions, plants have evolved complex and efficient antioxidant defense systems, including enzymatic and non-enzymatic antioxidant systems, over a long evolutionary period [4]. The non-enzymatic antioxidant chemistry system is primarily composed of antioxidants, such as ascorbate (AsA), glutathione (GSH), α-tocopherol, phenolic compounds, flavonoids, and alkaloids. The enzymatic antioxidant system is primarily composed of superoxide dismutase (SOD), catalase (CAT), peroxidase (POX), ascorbate peroxidase (APX), monodehydroascorbate reductase (MDHAR), glutathione reductase (GR), glutathione peroxidase (GPX), and other antioxidant enzymes [4,8]. These enzymatic and non-enzymatic antioxidant system members cooperate to efficiently scavenge ROS, protecting cells from damage in adverse environments. In plant cells, chloroplasts, peroxisomes, and mitochondria are the major sites for ROS production [5], and each site is equipped with an array of antioxidant systems that can efficiently scavenge redundant ROS. CATs are the major scavengers of peroxisomal H_2_O_2_, effectively catalyzing its degradation into harmless H_2_O and O_2_ [9]. CAT enzymes are encoded by a small gene family and are widely distributed among plants. Multiple studies have revealed that different plant species contain various numbers of *CAT* gene families, e.g., there are three *AtCAT* genes in *Arabidopsis thaliana* [10,11], three *OsCAT* genes in rice (*Oryza sativa*) [10,12], three *RsCAT* genes in small radish (*Rhaphanus sativus*) [13], four *CsCAT* genes in cucumber (*Cucumis sativus*) [14], four *GmCAT* genes in soybeans (*Glycine max*) [15], seven *GhCAT* genes in cotton (*Gossypium hirsutum*) [16], ten *TaCAT* genes in wheat (*Triticum aestivum*) [17], fourteen *BnCAT* genes in rapeseed (*Brassica napus*) [18], and sixteen *SsCAT* genes in sugarcane (*Saccharum spontaneum*) [19].

Various studies have suggested that *CAT* plays a vital role in plant growth, development, and defense against adverse environmental stresses [20,21,22]. In *Arabidopsis*, *AtCAT1* is involved in scavenging H_2_O_2_, which is produced under low temperatures, drought, oxidative stress (3-aminotriazol, 3-AT), methyl viologen (MV), and H_2_O_2_ stresses [11]. It has been shown that *AtCAT2* is significantly induced by cold, drought, and bright-light stress treatments. Additionally, *AtCAT3* was shown to be significantly upregulated under abscisic acid (ABA) and oxidative treatments and significantly induced during the senescence stage [11]. Under normal conditions, the catalase activity in *AtCAT2* mutant leaves was only 20% of that of the wild-type and more H_2_O_2_ was accumulated compared to the wild-type; furthermore, the sensitivity of the *AtCAT2* mutant to H_2_O_2_, NaCl, cold, and bright-light stresses increased [23]. In another study, the results showed that the catalase activity in the *AtCAT2* mutant leaves was only 10% of that of the wild-type, and *AtCAT3* mutants exhibited an approximate 20% reduction in catalase activity, while *AtCAT1* mutants showed similar catalase activity to the wild-type. Double *AtCAT1 AtCAT2* and *AtCAT2 AtCAT3* mutants showed a similar decrease in leaf catalase activity compared to *AtCAT2* mutants [24]. In rice, *OsCATCs* can be phosphorylated and activated by *STRK1*, which regulates H_2_O_2_ homeostasis and enhances tolerance to salt and oxidation stresses [25]. A recent study showed that the *OsCAT3* prokaryotically expressed protein can significantly remove H_2_O_2_ [12]. Additionally, *OsCAT3* knockout plants showed low viability and significantly reduced CAT, POD, and SOD enzyme activities [12].

Natural rubber (cis-1,4-polyisoprene) represents an important industrial raw and strategic material for people’s welfare, defense security, and public safety [26]. Due to its unique physical and chemical properties, including elasticity, abrasion resistance, impact, effective thermal dispersion, and ductility at low temperatures [27,28], it has the irreplaceable properties of synthetic rubber, especially in the military, deep sea, and aerospace industries. The rubber tree (*Hevea brasiliensis*) is a major typical tropical tree species that is capable of producing commercial sources of natural rubber [29], accounting for more than 98% of the world’s natural rubber production [30]. Currently, rubber trees are cultivated in more than 40 countries around the world [31], with more than 90% of the natural rubber production taking place in South and Southeast Asia [26,27,28,29]. With the escalating demand for natural rubber, rubber tree cultivation plantations have progressively expanded to nontraditional regions, such as the central highlands of Vietnam (12° N), northern India (29° N), the southern highlands of Brazil (23° S), and northeastern Thailand, as well as southwestern China (22° N) [32]. Consequently, commercial rubber tree plantations are frequently exposed to various environmental stresses (drought, cold, strong wind), over-tapping, and overstimulation, which can affect the growth and development of rubber tree, ultimately leading to a decline in production [33,34,35].

To the best of our knowledge, the *CAT* gene family has not been characterized in rubber tree. Therefore, a comprehensive genome-wide analysis of the *CAT* gene family in rubber tree was performed, and the physicochemical properties, gene structures, protein motifs, chromosomal locations, phylogenetic tree, cis-elements, gene duplication, and homology analyses were discussed in detail. Furthermore, the expression patterns of *HbCAT* genes under various abiotic stresses and exogenous hormone treatments were comprehensively analyzed.

## 2. Results

### 2.1. Identification of the CAT Gene Family in Rubber Tree

In the present study, five *CAT* genes were identified in the complete genome of the rubber tree via HMMER search and BLASTP methods. Detailed information on the five *HbCAT* genes is presented in Table 1. These genes are named *HbCAT1*–*HbCAT5* according to their order of localization on the chromosomes. Further analysis indicated that the coding sequence (CDS) lengths of these *HbCAT* genes ranged from 1236 bp (*HbCAT5*) to 1704 bp (*HbCAT2*), and the lengths of the encoded protein sequences ranged from 411 aa (*HbCAT5*) to 567 aa (*HbCAT2*). The molecular weights ranged from 47.15 kDa (*HbCAT4*) to 65.13 kDa (*HbCAT2*), and the isoelectric points ranged from 6.45 (*HbCAT4*) to 7.60 (*HbCAT1*).

### 2.2. Phylogenetic Analysis of CAT Genes

**Figure 1:**
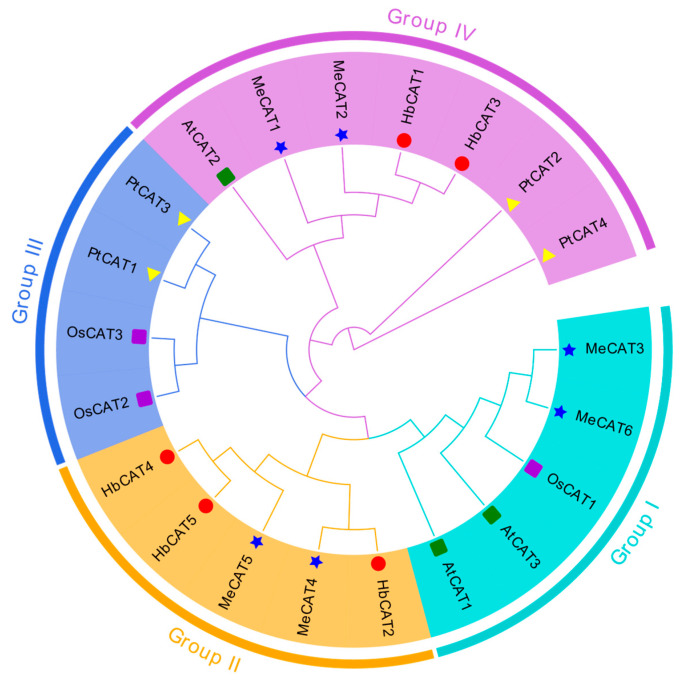
Phylogenetic analysis of CAT proteins from *H. brasiliensis*, *A. thaliana*, *O. sativa*, *P. trichocarpa*, and *M. esculenta*. The sequences of five HbCAT proteins (red circle), three AtCAT proteins (green rectangles), three OsCAT proteins (dark-violet rectangles), four PtCAT proteins (yellow triangles), and six MeCAT proteins (blue stars) were multiply aligned using Clustal W. The phylogenetic tree was constructed using the neighbor-joining (NJ) method with 1000 bootstrap replications. Four subgroups (Groups I–IV) were classified with different background colors.

To investigate the phylogenetic relationships of *HbCAT* genes between other plant species, we obtained three *AtCAT* genes from a dicotyledonous model plant the *Arabidopsis* (*Arabidopsis thaliana*) database. Additionally, we identified Os*CAT*, *PtCATs*, and *MeCATs* from monocotyledonous model plant rice (*Oryza sativa*), woody model plant populus (*Populus trichocarpa*) [36,37,38], and euphorbiaceae family cassava (*Manihot esculenta*) genomes using the same methods (HMMER and BLASTP). Three *CAT* genes were identified in rice, four in populus, and six in cassava (Table S2). A phylogenetic tree was constructed using the neighbor-joining (NJ) method based on the alignment of 21 CAT protein sequences, which were classified into four groups (Groups I–IV) (Figure 1). As shown in Figure 1, five *CAT* members (*AtCAT1*, *AtCAT3*, *OsCAT1*, *MeCAT3*, and *MeCAT6*) were clustered in group I; five *CAT* members (*HbCAT2*, *HbCAT4*, *HbCAT5*, *MeCAT4*, and *MeCAT5*) were clustered in group II; four *CAT* members (*OsCAT2*, *OsCAT3*, *PtCAT1*, and *PtCAT3*) were clustered in group III; and seven *CAT* members (*AtCAT2*, *MeCAT1*, *MeCAT2*, *HbCAT1*, *HbCAT3*, *PtCAT2*, and *PtCAT4*) were clustered in group IV. Furthermore, *HbCAT* genes were mainly clustered into two evolutionary branches (groups II and IV), and group II represented the unique branch of the euphorbiaceous family (Figure 1).

### 2.3. Gene Structures and Conserved Motifs of CATs

**Figure 2:**
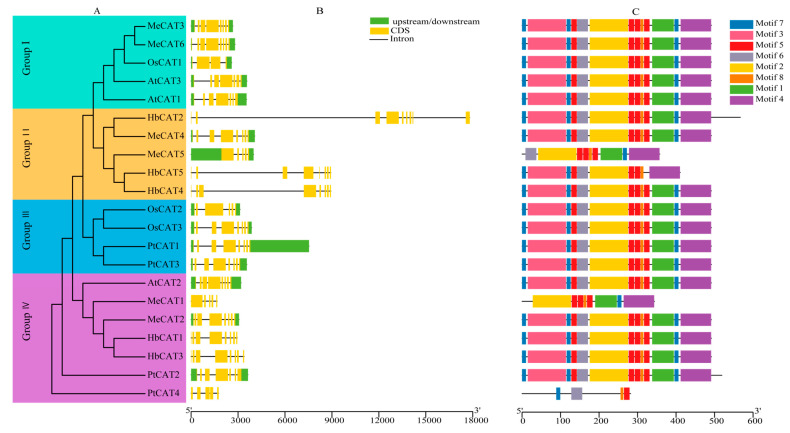
Phylogenetic tree, gene structures, and conserved motifs of the *CAT* genes from *H. brasiliensis*, *A. thaliana*, *O. sativa*, *P. trichocarpa*, and *M. esculenta*. (**A**) A phylogenetic tree was constructed using the neighbor-joining (NJ) method with 1,000 bootstrap replications. (**B**) Gene structure of *CAT* genes. Upstream/downstream sequences are represented by light green boxes, and exons and introns are represented by yellow boxes and black horizontal lines, respectively. (**C**) Conserved motifs of CAT proteins. Different colored boxes show various motifs.

To better understand the *HbCAT* gene family, we extracted the information on the *CAT* gene structures from the genome annotation file and visualized them using TBtools software (v1.0987663) (Figure 2B). We observed that all *HbCAT* genes exhibited a consistent pattern of eight exons and seven introns, except for *HbCAT2*, which had nine exons and eight introns. Furthermore, we found that the intron-exon structural patterns of *CAT* genes are highly conserved across species. For example, 12 (57.14%) out of the 21 *CAT* genes contained eight exons and seven introns. To further explore the functional diversity of *CAT* genes, conserved motifs of CAT proteins were analyzed using the MEME online website, and eight motifs were identified (Figure 2C). Detailed information on the eight motifs is presented in supplementary Table S3 and Figure S1. Almost all *CAT* genes from different species contained eight conserved motifs, except *MeCAT5* (7), *HbCAT5* (7), *MeCAT1* (6), and *PtCAT4* (4). In conclusion, *CAT* genes from different species have highly similar conserved gene structures and motif patterns, and the ones on the same evolutionary branch have similar gene structures and conserved motif patterns. These results strongly support evolutionary taxonomic identity and suggest that *CAT* genes are conserved during evolution (Figure 2A).

**Figure 3:**
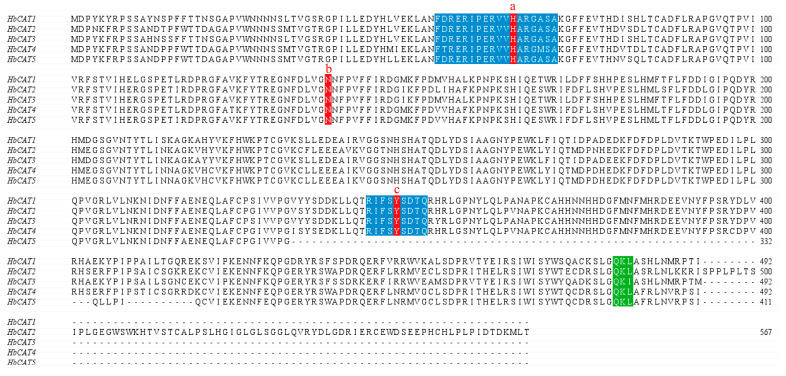
Multiple protein sequence alignment of the *HbCAT* gene family. Three conserved amino acids, His (**a**), Asn (**b**), and Tyr (**c**), are highlighted in red. The active catalytic site (FDRERIPERVVHARGASA) and the heme-ligand signature sequence (RLFSYNDTQ) are highlighted in blue, and the peroxisome-targeting signal 1 (PTS1) sequence is highlighted in green.

A multiple protein sequence alignment analysis indicated that except for the *HbCAT5* gene, which has no heme-ligand signature sequence (RIFSYSDTQ) and tyrosine (Tyr), all *HbCAT* genes contained one active catalytic site (FDRERIPERVVHARGASA), one heme ligand signature sequence (RIFSYSDTQ), and three conserved amino acid residues, such as histidine (His), tyrosine (Tyr), and aspartic acid (Asn). In addition, all proteins contained one peroxisome targeting signal type 1 (PTS1) sequence (QKL) (Figure 3).

### 2.4. Chromosomal Localization and Synteny Analysis of CAT Genes

**Figure 4:**
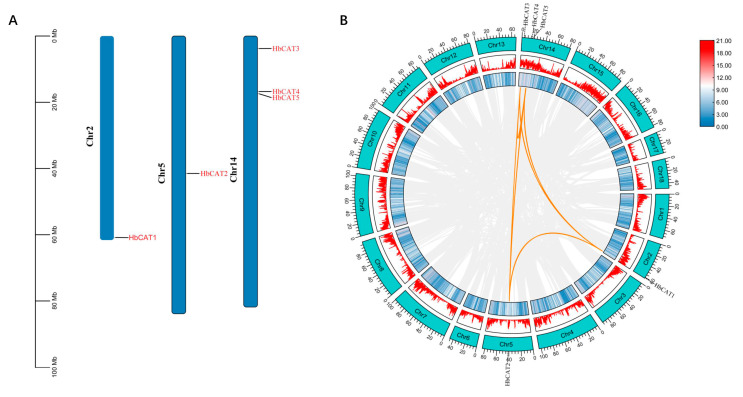
Chromosomal localization and replication events of *HbCAT* genes. (**A**) Chromosomal distribution of the five *HbCAT* genes. The scale on the left side of the chromosome refers to the location of the genes in *H. brasiliensis*. Chromosome numbers (Chr2, Chr5, and Chr14) are shown on the left of the chromosomes. (**B**) Circle map of duplicated gene pairs for *HbCAT* genes. The heat map and rectangular histogram with blue lines indicate gene density on the chromosomes. The orange indicates segment duplicated *HbCAT* gene pairs, while the gray line presents all the syntenic blocks in the *H. brasiliensis* genome.

Chromosomal location analysis indicated that five *HbCAT* genes were unevenly anchored to only three of the eighteen chromosomes of the rubber tree. *HbCAT1* and *HbCAT2* were located on Chr2 and Chr5, respectively, and *HbCAT3*, *HbCAT4*, and *HbCAT5* were located on Chr14 (Figure 4A).

To better understand the evolution of *HbCAT* genes, replication events of *HbCAT* genes were analyzed using the TBtools software (v1.0987663) [39]. As shown in Figure 4B, there were eight homologous *HbCAT* gene pairs in the rubber tree, originating from fragment replication, which included *HbCAT1/HbCAT2*, *HbCAT1/HbCAT3*, *HbCAT1/HbCAT4*, *HbCAT2/HbCAT4*, *HbCAT2/HbCAT3*, *HbCAT2/HbCAT5*, *HbCAT3/HbCAT4*, *and HbCAT4/HbCAT5* (Figure 4B, Table 2). Although the *HbCAT3*, *HbCAT4*, and *HbCAT5* genes were located on the same chromosome (Chr14), they were separated by at least one megabase (Table 2), suggesting that these genes may have been formed by fragmentary replication. In conclusion, these results suggest that the amplification of the *HbCAT* gene family was primarily derived from fragment replication. The Ka, Ks, and Ka/Ks values of *HbCAT* homologous gene pairs were evaluated, and the results showed that the Ka/Ks values of all homologous gene pairs were less than 1 (Table 2), indicating that purifying selection is the selective pressure for *HbCAT* gene evolution.

**Figure 5:**
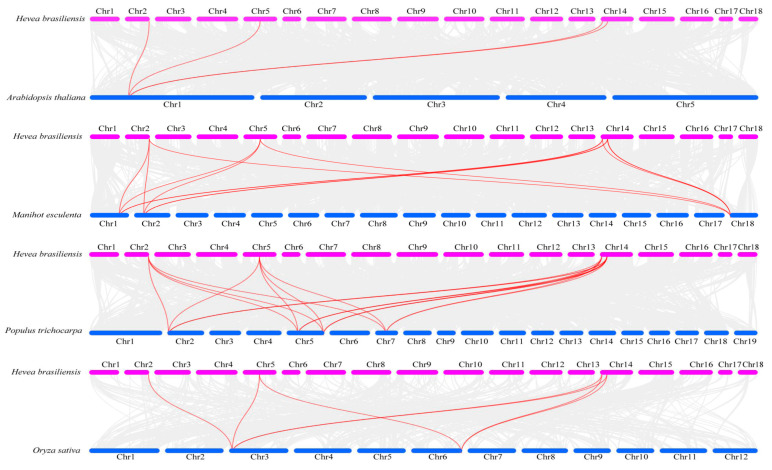
Synteny analysis of *HbCAT* genes between *AtCATs*, *OsCATs*, *PtCATs,* and *MeCATs*. Gray lines in the background represent all collinearity blocks in the genomes of different species, and red lines represent syntenic *CAT* gene pairs.

To further investigate the evolutionary origins and orthologous relationships of *CAT* genes among different plant species, a synteny analysis of four dicotyledonous plants (*H. brasiliensis, P. trichocarpa*, *A. thaliana*, and *M. esculenta*) and one monocotyledonous plant (*O. sativa*) was performed. As shown in Figure 5, there are four orthologous *CAT* gene pairs between *H. brasiliensis* and *A. thaliana*, nineteen pairs between *H. brasiliensis* and *P. trichocarpa,* thirteen pairs between *H. brasiliensis* and *M. esculenta*, and seven pairs between *H. brasiliensis* and *O. sativa*. Four *HbCAT* genes (*HbCAT1*, *HbCAT2*, *HbCAT3*, and *HbCAT4*) had homologous gene pairs for *O. sativa*, *A. thaliana*, *P. trichocarpa*, and *M. esculenta*, indicating that these genes possibly evolved from genes that existed before the differentiation of monocotyledons and dicotyledons. Homologous gene pairs of the *HbCAT5* gene were present in woody plants, such as *P. trichocarpa* (*HbCAT5/PtCAT1/2/3*) and *M. esculenta* (*HbCAT5/MeCAT5*), but not in the herbaceous plants *A. thaliana* and *O. sativa* (Table S4), suggesting that *HbCAT5* may have formed after the differentiation of herbaceous and woody plants.

### 2.5. Analysis of Cis-Elements in the Promoters of HbCAT Genes

**Figure 6:**
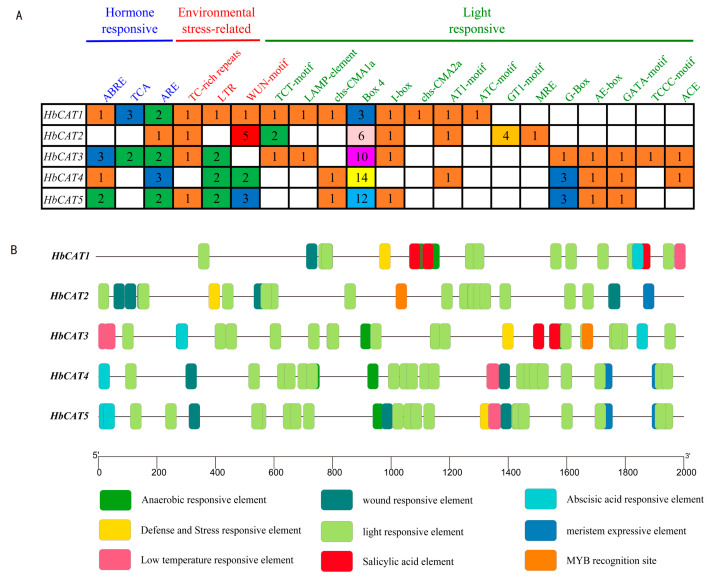
Cis-acting elements in the promoter region of *HbCAT* genes. (**A**) Classification and statistical analysis of cis-regulatory elements. The number in the colored rectangles indicates the number of cis-acting elements. (**B**) The distribution of cis-acting elements in the *HbCAT* promoter region (−2000 bp). Different colored rectangles indicates different cis-elements.

To further understand the potential regulatory mechanisms of the *HbCAT* genes under abiotic stresses, cis-acting elements in the promoter regions of each *HbCAT* gene were scanned using the plantCARE. A total of 25 cis-acting elements were detected in the promoter regions of the *HbCAT* genes, which were mainly categorized into four categories: hormone-responsive, environmental stress-related, organizational development, and light-responsive. Detailed information on these cis-acting elements is presented in Table S5. Hormone-responsive elements were identified in several *HbCAT* genes (Figure 6A,B), e.g., the abscisic acid (ABA) response element ABRE was detected in the *HbCAT1/3/5* genes, and the salicylic acid (SA) response element TCA was identified in the *HbCAT1/3* genes, suggesting that the expression of these genes may be regulated by the ABA and SA hormone signaling pathways. In addition, at least one abiotic stress-related responsive element was identified in almost all *HbCAT* genes. For example, *HbCAT1/2/3/5* contained defense and stress-responsive elements (TC-rich repeats), *HbCAT1/3/4/5* contained LTR elements involved in low-temperature responsiveness, and *HbCAT1/2/4/5* contained wound-responsive elements (WUN motifs), suggesting that these genes may be involved in the response to various abiotic stresses. *HbCAT2/4/5* contained a cis-acting regulatory element related to meristem expression, implying that these genes may be involved in meristem development. *HbCAT2* and *HbCAT3* contained MYB transcription factor-binding sites (Figure 6B), suggesting that the *HbCAT2* and *HbCAT3* genes may be regulated by MYB transcription factors. Moreover, almost all *HbCAT* genes contained various light-responsive elements (TCT-motif, LAMP-element, chs-CMA1a, Box 4, I-box, chs-CMA2a, AT1-motif, ATC-motif, GT1-motif, MRE, G-Box, AE-box, GATA-motif, TCCC-motif, and ACE), and Box4 and G-box elements were abundantly enriched (Figure 6A,B; Table S5), suggesting that *HbCAT* genes may play vital roles in light stress response.

### 2.6. Functional Gene Ontology (GO) Annotation of HbCAT Genes

**Figure 7:**
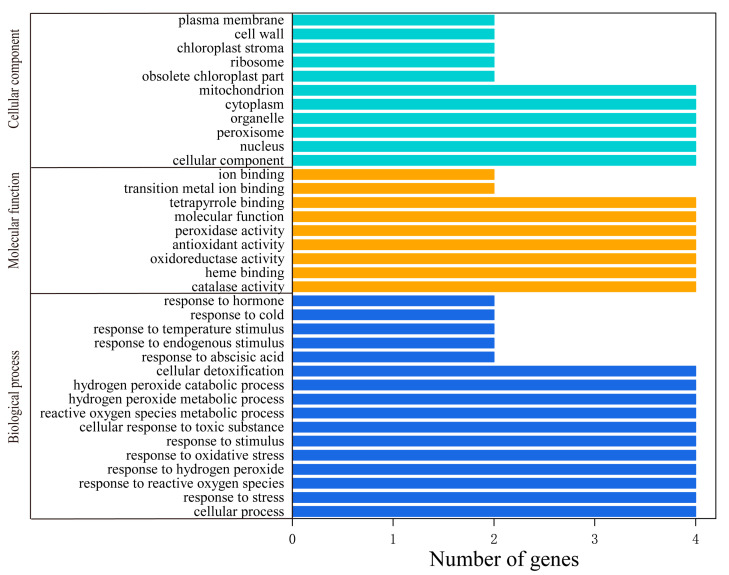
GO enrichment analysis of *HbCAT* in rubber tree.

To further understand the potential functions of the *HbCAT* genes, we performed a gene ontology (GO) annotation and enrichment analysis. The GO_BP enrichment analysis showed that these genes were primarily involved in responding to abiotic and oxidative stresses, e.g., response to reactive oxygen species (GO:0000302), stress (GO:0006950), hydrogen peroxide (GO:0042542), oxidative stress (GO:0006979), stimulus (GO:0050896), cellular response to toxic substances (GO:0097237), reactive oxygen species metabolic process (GO:0072593), hydrogen peroxide metabolic process (GO:0042743), hydrogen peroxide catabolic process (GO:0042744), and cellular detoxification (GO:1990748) (Figure 7; Table S6). Several genes were involved in responding to hormones, e.g., *HbCAT4/5* in response to abscisic acid (GO:0009737) (Figure 7; Table S6). The GO_MF enrichment analysis indicated that these genes were primarily involved in catalase activity (GO:0004096), oxidoreductase activity (GO:0016491), antioxidant activity (GO:0016209), peroxidase activity (GO:0004601), heme binding (GO:0020037), tetrapyrrole binding (GO:0046906), and metal ion binding (GO:0046872) (Figure 7; Table S6). The GO_CC enrichment analysis indicated that these genes were primarily enriched in the nucleus (GO:0005634), peroxisome (GO:0005777), organelles (GO:0043226), cytoplasm (GO:0005737), and mitochondria (GO:0005739). These results are consistent with the predicted subcellular localization of the CAT proteins (Figure 7; Table S6).

### 2.7. Expression Patterns of HbCAT Genes in Different Tissues

**Figure 8:**
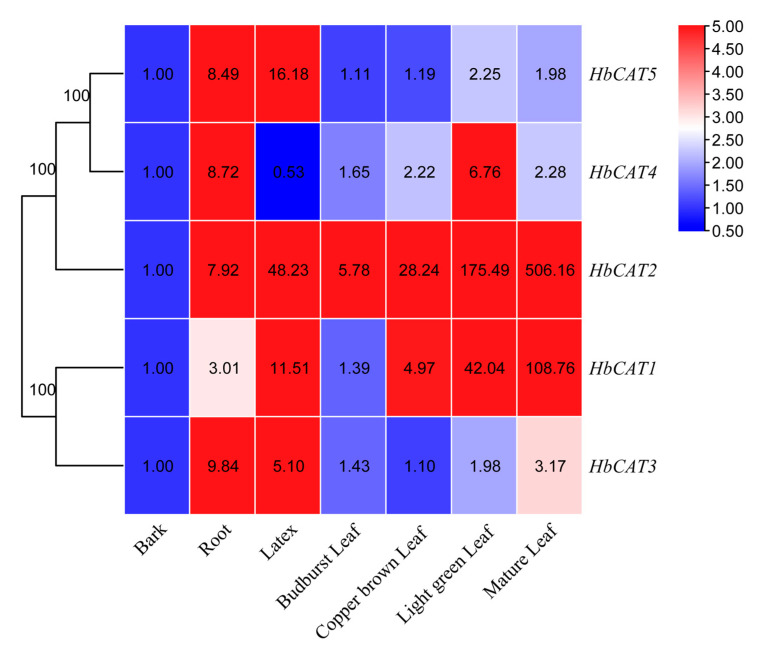
Expression profiles of *HbCAT* genes in different tissues (bark, root, latex, budburst leaf, copper brown leaf, light green leaf, and mature leaf). The color bar shows the relative expression levels of the genes calculated based on the bark.

The expression patterns of the *HbCAT* genes in seven different tissues were detected using the RT-qPCR technique to elucidate their transcript levels in various tissues. As shown in Figure 8, *HbCAT* genes were constitutively expressed in the bark, root, latex, budburst leaf, copper-brown leaf, light-green leaf, and mature leaf of the rubber tree cultivar GT1, albeit at different levels. For instance, *HbCAT1* and *HbCAT2* were highly expressed in all tissues. All *HbCAT* genes showed high expression levels in the root and latex, except for *HbCAT4*, which exhibited a low level in latex. The expression levels of all *HbCAT* genes gradually increased with leaf development and remained on a higher level in mature leaves compared with the other stages, suggesting that these genes may play vital roles in the growth and development of rubber tree leaves.

### 2.8. Expression Patterns of HbCAT Genes under Different Abiotic Stresses and Exogenous Hormone Treatments

**Figure 9:**
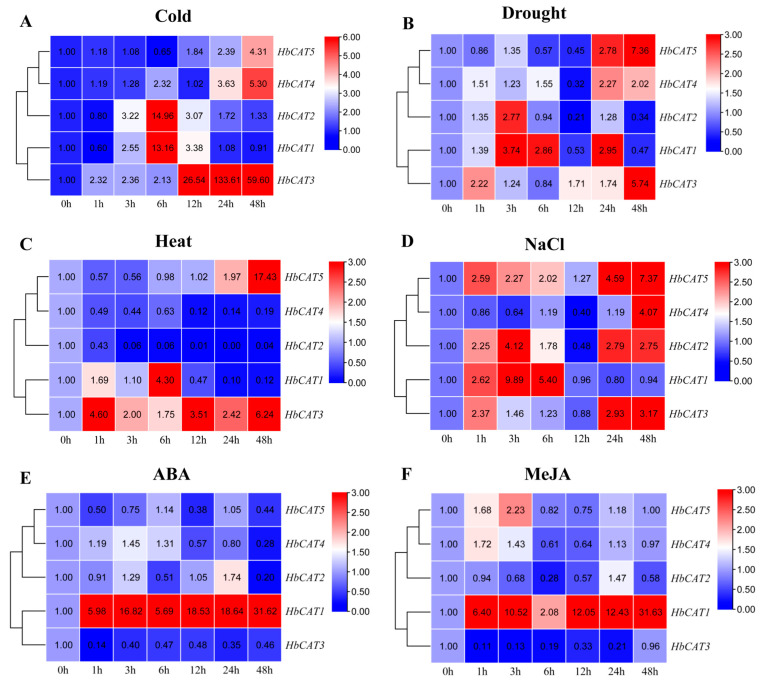
Expression patterns of the *HbCAT* genes under different abiotic stresses and exogenous ABA and MeJA treatments (**A**–**F**). Color bars represent different expression levels ranging from low levels (blue) to high levels (red). Numbers represent the relative expression levels of *HbCAT* genes computed based on the expression level at 0 h and the mean of three replicates.

To confirm whether the *HbCAT* genes responded to different abiotic stresses and exogenous hormone treatments, the expression patterns of the *HbCAT* genes under low temperature (4 °C), high temperature (42 °C), drought, and high salt (300 mM NaCl) stresses were analyzed using the RT-qPCR technique. The results indicated that the expression levels of most of the *HbCAT* genes were significantly induced by most of the treatments (Figure 9). For instance, the expression level of *HbCAT3* increased gradually with the duration of cold stress and peaked after 24 h, which was 133.06-fold that of the control (Figure 9A). *HbCAT3* was also strongly upregulated under high temperature, drought, and NaCl treatments and peaked after 48 h (Figure 9B,D). The expression levels of *HbCAT1/2* initially increased and then decreased under cold, drought, and salt stresses. In contrast, *HbCAT4/5* genes were not significantly altered at early treatment stages (1 h, 3 h, 6 h, and 12 h); however, they were significantly upregulated at the late treatment stages (24 h and 48 h) under cold and drought stresses (Figure 9A,B,D). Under heat stress, the expression levels of *HbCAT2/4* were significantly downregulated at all time points during the treatment (Figure 9C). The expression level of *HbCAT1* was strongly induced by ABA and MeJA and significantly upregulated at all time points during the treatments. In contrast, *HbCAT3* was strongly downregulated under ABA and MeJA treatments (Figure 9E,F).

## 3. Discussion

Abiotic stress is a vital factor affecting rubber tree growth, development, latex yield, and latex quality [40,41,42], especially in China, which is located in a nontraditional rubber plantation belt. Catalase (CAT) has been confirmed to play a vital role in plant abiotic stresses [43,44,45]. However, the characterization of the *HbCAT* gene family of rubber tree and its biological function under abiotic stress has not been reported. In our study, a total of five *HbCAT* genes were identified in rubber tree based on a genome-wide search using catalase (PF00199) and catalase-related (PF06628) Hidden Markov Model (HMM) files as references and comparative analyses with AtCAT protein sequences, which were localized on chromosomes Chr2 (*HbCAT1*), Chr5 (*HbCAT2*), and Chr14 (*HbCAT3/4/5*) (Figure 4A). We identified four *PtCAT* gene family members in *P. trichocarpa* and six *MeCAT* gene family members in *M. esculenta*, using the same methods (HMMER and BLASTP). Previous studies have also shown that the number of *CAT* gene families is small in plants, e.g., there are three *CAT* genes in *A. thaliana* [11], rice [10,12], and small radish [13], four in cucumber and soybeans [14,15], seven in cotton [16], ten in wheat [17], fourteen in rapeseed [18], and sixteen in sugarcane [19]. Typical CAT protein is a tetrameric enzyme with four identical subunits, and each subunit contains a heme prosthetic group in the catalytic center [46]. In this study, we found that the *HbCAT* (*HbCAT1*-*HbCAT4*) genes had one active catalytic site (FDRERIPERVVHARGASA), one heme ligand signature motif (RIFSYSDTQ), and three conserved amino acid residues (HIS, Tyr, and Asn) (Figure 3). In addition, all *HbCAT* (*HbCAT1*-*HbCAT5*) genes included one peroxisome targeting signal type 1 (PTS1) sequence (QKL). These results are consistent with previous research on cucumber and sugarcane [14,19]. Peroxisomal matrix proteins primarily interact with the cytoplasmic receptor PEX5 via the C-terminal tripeptide of PTS1, thereby causing efficient transport to the peroxisome [47,48]. In pumpkin (*Cucumis sativus*), *CAT1* contains a C-terminal PTS1 (peroxisome-targeting signal 1) sequence QKL, which deviates from the conventional SKL motif of typical PTS1 signals [49]. In *Arabidopsis*, the import of *CAT1* into peroxisomes relies on its dependence on the cytoplasmic PTS receptor PEX 5p, which is similar to typical PTS1 import; however, unlike typical PTS1, *CAT1* specifically interacts with the N-terminal structural domain of PEX 5 rather than its C-terminal structural domain [50]. Although the precise mechanism of plant CAT entry into the peroxisome remains unclear, it is noteworthy that the C-terminal PST1 sequence plays a pivotal role in this process. These results indicated that almost all members of the *HbCAT* gene family have conserved CAT structural domains and exert functions related to CAT activity.

According to the phylogenetic tree analysis, 21 *CATs* were clustered into four groups, which is consistent with previous research on *CAT* evolutionary relationships between the *BnCAT*, *BoCAT*, *BraCAT*, and *AtCAT* genes [18]. *HbCATs* were primarily clustered into groups II and IV along with *AtCAT*s and *MeCAT*s (Figure 1), suggesting that the evolutionary pedigrees of rubber tree, *Arabidopsis*, and cassava are comparable. Notably, group II contained only *HbCAT* and *MeCAT* genes, implying that these genes may be unique to euphorbiaceous plants and have distinct biological functions. The study of gene intron-exon structural patterns can contribute to our understanding of the phylogenetic history of the *CAT* gene family. The gene exon-intron structure represents a vital feature of gene evolution. In general, closely related genes have similar exon-intron patterns and protein structures [51,52]. In our study, all *HaCAT* genes contained seven introns and eight exons, except *HbCAT2*, which had eight introns and nine exons (Figure 2B), suggesting that the number of introns and exons of *HbCAT* genes has not changed significantly during the evolutionary process. *CAT* genes from *H. brasiliensis*, *A. thaliana*, *O. sativa*, *P. trichocarpa*, and *M. esculenta* showed highly conserved structures. For example, 12 (57.14%) of the 21 *CAT* genes contained seven introns and eight exons. A previous study reported that the ancestral copy of the *CAT* gene included seven introns, and these positions are conserved [53]. In addition, all HbCAT proteins contained catalase (PF00199) and catalase-related (PF06628) domains, and together these results confirmed that *CAT* genes are highly conserved during plant evolution, with a few exceptions. Similar findings were obtained in several previous studies [17,18].

Replication events are the basis for the divergence of homologous gene functions and the primary driver of gene family membership expansion [54], which is the most assessed method in gene family expansion analyses. In the present study, gene duplication event analysis suggested that fragment duplication was the primary driver of *HbCAT* gene evolution. Numerous studies have reported that segmental duplication was the primary driving force of expansion for *CAT* gene family members [15,19]. Evaluation of the Ka, Ks, and Ka/Ks values for *HbCAT* homologous gene pairs showed that the purification selection is a selection pressure for *HbCAT* gene evolution (Table 2). Meanwhile, to investigate the evolutionary relationship of *HbCAT*s, we performed a synteny analysis of *HbCAT* genes between *AtCATs*, *OsCATs*, *PtCATs*, and *MeCATs*. The results showed that the highest to lowest homologous pairs were populus (19), cassava (13), rice (7), and *Arabidopsis* (4) (Table S4). Interestingly, *HbCAT5* had no homologous pairs with rice and *Arabidopsis*; however, it had three and one homologous gene pairs with populus and cassava, respectively, which may be unique to woody plants, and this gene may have an irreplaceable function in plants.

Cis-acting elements play a significant regulatory role in gene transcription and can bind sites for transcription factors with core promoters and regulate the transcription of target genes [55,56]. Previous studies have suggested that *CAT* promoters contain numerous light-, hormone-, stress-responsive cis-acting elements, meristem expression relevant elements, and MYB binding sites. Furthermore, CAT genes can be induced by different abiotic stresses, such as cold [17,18,19], drought [11,14,15,18,19], heat [57,58], and salt [14,17,18] stresses. In our study, promoter analyses revealed that all five *HbCAT* genes contained the core components, such as the “TATA box”, and a series of stress-related elements (TC-rich repeats, LTR elements, and WUN-motif), light-responsive elements (TCT-motif, LAMP-element, chs-CMA1a, Box 4, I-box, chs-CMA2a, AT1-motif, ATC-motif, GT1-motif, MRE, G-Box, AE-box, GATA-motif, TCCC-motif, and ACE), hormone-related cis-acting elements (ABRE, TCA, and ARE) (Figure 6A,B), and several genes (*HbCAT2*, and *HbCAT3*) contained MYB binding sites. Moreover, these cis-acting elements were irregularly dispersed in the promoter region of the *HbCAT* genes (Figure 6). Cis-acting elements distributed in different locations facilitate the regulation of protein functions under various environmental stimuli. These results suggested that *HbCAT* genes may be involved in the regulation of various stresses and biological processes.

Numerous studies have suggested that *CAT* genes play a vital role in plant growth, development, and defense against adverse environmental stresses. In *Arabidopsis*, the expression of *AtCAT1* was low under normal conditions; however, it was significantly upregulated by cold, drought, and oxidative stresses (H_2_O_2_, 3-aminotriazol, and methyl viologen) and by treatments with the plant hormones SA and ABA; *AtCAT2* was significantly upregulated under drought and H_2_O_2_ stresses and *AtCAT3* was significantly increased under oxidative stress and ABA treatments [11]. Further studies found that *AtCAT2* mutants exhibited enhanced sensitivity to H_2_O_2_, NaCl, cold, and bright light stresses [23]. In sugarcane, the expression of *ScCAT1* was significantly upregulated under *S. scitamineum* stress. Furthermore, overexpression of the *ScCAT1* gene in tobacco could enhance resistance to pathogen infection by scavenging excessively toxic ROS [19]. In durum wheat, the expression of *TaCAT2* and *TaCAT3* was significantly upregulated after salt, mannitol, cold, heat, and ABA treatments [59]. In soybean, the expression of the *GmaxCAT17.1* gene under drought stress exhibited a continuous upregulation at all treatment points (4 h, 8 h, and 12 h), and *GmaCAT6.1*, *GmaCAT4*, and *GmaCAT17.1* were significantly upregulated under combined drought and heat stresses [15]. In rapeseed, Raza et al. [18] reported that *BnCAT1*, *BnCAT2*, *BnCAT3*, *BnCAT12*, and *BnCAT13* were primarily induced by ABA, gibberellic acid (GA), IAA, cold, and salinity treatments. In our study, the expression of the *HbCAT3* gene was strongly upregulated by low temperature (4 °C), high temperature (42 °C), drought, and high salt (300 mM NaCl) stresses, and by ABA (100 µM) and MeJA (100 µM) treatments (Figure 9). The expression levels of the *HbCAT1/2* initially increased and then decreased under cold, drought, and salt stresses. In contrast, the expression levels of the *HbCAT4/5* genes were not significantly changed at early treatment stages (1 h, 3 h, 6 h, and 12 h); however, they were significantly upregulated at the late treatment stages (24 h and 48 h) under cold and drought stresses (Figure 9A,B,D). The expression level of *HbCAT1* was significantly upregulated at all time points under ABA and MeJA treatments. In contrast, the expression level of *HbCAT3* was strongly downregulated (Figure 9E,F), indicating a close correlation between the expression of these *HbCAT* genes and the ABA and MeJA signaling pathways in rubber tree. These findings suggest that *HbCAT* genes play crucial roles in responding to cold, heat, drought, and salt stress. CATs are key enzymes responsible for regulating cellular H_2_O_2_ levels to protect normal physiological processes from damage under abiotic stresses [9]. Wu et al. [19] found that the expression of the sugarcane *ScCAT1* gene exhibited a “rising and then declining” pattern in response to *S. scitamineum* stress in both YT93-159 (smut resistant) and ROC22 (smut susceptible) sugarcane cultivars. However, the CAT enzyme activity exhibited a sustained increase, suggesting that each member of the *CAT* gene family may make distinct contributions to the activity of catalase. Consequently, the relationship between *HbCAT* gene expression and CAT activity under abiotic stresses requires further investigation. In summary, these results lay the foundation for further exploration of the functions of stress-related *CAT* genes and further understanding of the roles of these genes in stress-tolerance mechanisms.

## 4. Materials and Methods

### 4.1. Identification of HbCAT Genes in Rubber Tree

The whole-genome protein sequence, nucleotide, and annotation files of the rubber tree [60] were downloaded from the NCBI database (https://www.ncbi.nlm.nih.gov, accessed on 2 March 2021) to identify the members of the *HbCAT* gene family in the rubber tree. Catalase (PF00199) and catalase-related (PF06628) Hidden Markov Model (HMM) files were obtained from the Pfam protein structural domain database (http://pfam.xfam.org/, accessed on 16 November 2021) [61]. The hmmsearch program of HMMER 3.0 software (http://www.eddylab.org/software/hmmer3/3.0/, accessed on 16 November 2021) [62] was used to scan the rubber tree protein data [63,64]. Protein sequences of three known *CAT* genes in *A. thaliana* (*AtCAT1*/AT1G20630, *AtCAT2*/AT4G35090, and *AtCAT3*/AT1G20620) were downloaded from the *Arabidopsis* genome database (https://www.arabidopsis.org/, accessed on 10 December 2021) [65] and used as reference sequences to blast the rubber tree protein data using the BLASTP method (version 2.2.6) [63,64]. We combined the results of the HMMER- and BlASTP-based searches and removed redundant sequences. The candidate sequences were further examined using the conserved structural domains (CDDs) online tool (https://www.ncbi.nlm.nih.gov/cdd, accessed on 11 May 2023) [66] and SMART database (http://smart.embl-heidelberg.de/, accessed on 11 May 2023) [67] to confirm the domain of the *CAT* gene family. Sequences without the conserved *CAT* domain were removed. The ProtParam tool (https://web.expasy.org/protparam/, accessed on 24 May 2023) on the ExPASy website [68] was used to predict the coding sequence (CDS) length, molecular weight (MW), and isoelectric points (pI). The WoLFPSORT tool (https://www.genscript.com/wolf-psort.html, accessed on 24 May 2021) [69] was used to predict subcellular localization.

### 4.2. Phylogenetic Tree, Gene Structures, and Conserved Motifs

To further clarify the evolutionary relationships between the *HbCATs* and *CATs* of other plant species, we downloaded the genome annotation and protein sequence files of the rice (*O. sativa* Japonica Group), Poplar (*P. trichocarpa*) [36,37,38], and cassava (*M. esculenta*) from the Ensembl Plant genome database (https://plants.ensembl.org/index.html, accessed on 5 March 2023) [70]. Similarly, HMMER searches based on catalase-conserved domains (PF00199 and PF06628) and BLASTP searches with AtCAT protein sequences as references were performed to obtain *CAT* gene families from different species. A neighbor-joining (NJ) phylogenetic tree was constructed using MEGA X [71] with 1000 independent replications, and the phylogenetic tree was further beautified using the Evolview online website (https://www.evolgenius.info/, accessed on 3 June 2023) [72]. Protein-conserved motifs were analyzed using the MEME website (http://meme-suite.org/tools/meme, accessed on 13 March 2023) [73], and the number of motifs was set to 8 and the sequence length to being between 6 bpm and 100 bp. Intron-exon information for *CAT* genes was extracted from the genome annotation file and visualized using TBtools software (v1.0987663) [39].

### 4.3. Chromosomal Localization, Gene Duplication, and Synteny Analysis

The chromosome location information of the *HbCAT* genes was obtained from the gene annotation file of the rubber tree genome, and each *HbCAT* gene was mapped to a chromosome using TBtools. The genome-wide replication events and replication relationships of *CATs* were analyzed using the MCScanX module of the TBtools software (v1.0987663) [39] with default values. Additionally, with the use of TBtools, the collinear relationships within the *HbCAT* gene family and between other species were visualized, and the nonsynonymous (ka)/synonymous (ks) substitutions of *CAT* gene pairs were identified.

### 4.4. Cis-Acting Elements Analysis

To further investigate the potential functions of *HbCAT* genes, we extracted 2000 bp DNA sequences upstream of the *HbCAT* gene start codon (ATG) as the promoter sequence of the *HbCAT* genes using TBtools software (v1.0987663) [39] and submitted these sequences to the PlantCARE online tool (http://bioinformatics.psb.ugent.be/webtools/plantcare/html/, accessed on 3 April 2023) [74] to predict potential cis-acting elements for each promoter region. Subsequently, the prediction results were visualized using TBtools.

### 4.5. GO Enrichment Analysis of HbCAT Genes

Gene functional annotation analysis was performed using the online eggNOG database (http://eggnog-mapper.embl.de/, accessed on 20 June 2023) [75]. The GO annotation-based package hierarchy file (go-basic.obo) was downloaded, followed by a GO functional enrichment analysis using the “GO enrichment” module of TBtools software (v1.0987663) [39].

### 4.6. Plant Materials and Stress Treatments

The 12-year-old rubber tree cultivar GT1 clone was used to determine the tissue-specific expression grown on the Experimental Farm of Yunnan Institute of Tropical Crop (100.788 E, 22.036 N). The trees were regularly tapped for latex collection using a half-spiral pattern every four days (S/2, d/4) without ethylene (ET) stimulation. Samples of leaves at different developmental stages (budburst, copper-brown, light-green, and mature stages), bark, root, and latex were collected from ten healthy tapping trees, with three biological replicates.

The expression patterns of the *HbCAT* gene family under various abiotic stresses and exogenous hormone (ABA, MeJA) treatments were analyzed using asexual GT1 bagged seedlings that had developed one extension unit, and the leaves were completely mature. Plantlets were transferred into a growth chamber under a 16 h light/8 h dark cycle at 28 °C with 80% relative humidity. Two days later, plantlets were treated in a growth chamber at 4 °C and 42 °C for low/high-temperature stress treatments. For the drought stress treatment group, plantlets were removed from all culture bags and soil, and bare-root cultivated in a growth chamber at 28 °C with an 80% relative humidity [63,76]. For the salt stress treatment, plantlets were irrigated with 300 mM NaCl solution [63]. For the hormone treatments, plantlets were sprayed with 100 µM abscisic acid (ABA) and 100 µM methyl jasmonate (MeJA) solution, and the control group was treated with 0.02% Tween-20. Leaf samples were collected at various time points (0, 1, 3, 6, 12, 24, and 48 h) during treatments and immediately frozen in liquid nitrogen and stored at −80 °C for further analysis. There were three independent biological replicates in each sample from five plantlets (*n* = 5).

### 4.7. RNA Extraction and RT-qPCR Analysis

Total RNA was extracted from each sample using an RNAprep Pure Plus kit (Tiangen, Beijing, China). First-strand cDNA was synthesized using a RevertAid TM First Strand cDNA Synthesis kit (Thermo Scientific, Vilnius, Lithuania). Specific primers were designed using the NCBI database (https://www.ncbi.nlm.nih.gov/, accessed on 29 April 2022) and synthesized by Sangon Biotech (Shanghai) Co., Ltd. (Shanghai, China). Detailed primer sequences are listed in Table S1. RT-qPCR analysis was performed using TB Green^®^ Premix Ex Taq™ (Takara, Beijing, China) from the TaKaRa Company and a qTOWER3G real-time fluorescence quantification system (Shanghai, China). The *HbActin7a* gene was used as an internal reference. Three technical replicates were performed for RT-qPCR. Raw data were normalized as described previously [63,76].

## 5. Conclusions

We identified five *HbCAT* genes from rubber tree, three *OsCAT* genes from rice, four *PtCAT* genes from poplar, and six *MeCAT* genes from cassava. The physicochemical properties, phylogenetic tree, gene structures, conserved motifs, chromosomal localization, gene duplications, collinear, cis-acting elements, and GO enrichment of the *HbCAT* genes were comprehensively analyzed. Furthermore, tissue-specific expression analysis suggested that *HbCAT* genes may play vital roles in the growth and development of rubber tree. RT-qPCR results indicated that *HbCAT*s play important roles in responding to cold, heat, drought, and NaCl abiotic stresses. Moreover, the expression of some *HbCAT* genes was regulated by ABA or MeJA hormones. To summarize, this study provides the basis for the functional characterization of *HbCATs* in growth, development, hormone response, and abiotic stress.

## Figures and Tables

**Table 1:** Detailed information on *HbCAT* genes identified in rubber tree.

Gene Name	Gene ID	Chr	Genomic Position (5′–3′)	CDS Length (bp)	Exon	Protein Length (aa)	Molecular Weight (kDa)	Isoelectric Point (pI)	Subcellular Localization
*HbCAT1*	GH714_028220	2	60,823,793–60,826,740 (+)	1479	8	492	56.75	7.60	Peroxisome
*HbCAT2*	GH714_016778	5	41,479,542–41,497,345 (−)	1704	9	567	65.13	6.84	Cytoplasm
*HbCAT3*	GH714_034861	14	3,781,996–3,785,382 (−)	1479	8	492	56.89	7.24	Cytoplasm
*HbCAT4*	GH714_007303	14	16,753,136–16,762,068 (−)	1479	8	492	47.15	6.45	Cytoplasm
*HbCAT5*	GH714_007170	14	17,423,940–17,432,871 (−)	1236	8	411	56.81	6.82	Cytoplasm

Hb: *H. brasiliensis*; CAT: catalase; (+) and (−) indicate genes on the positive or negative strand, respectively.

**Table 2:** The Ka and Ks values of *HbCAT* gene pairs in rubber tree.

Group A		Group B		Ks	Ka	Ka/Ks	Duplicated Type	Selection Pressure
Gene Name	Gene ID	Gene Name	Gene ID					
*HbCAT1*	GH714_028220	*HbCAT2*	GH714_016778	0.083	1.519	0.054	Segmental duplication	Purifying selection
*HbCAT1*	GH714_028220	*HbCAT3*	GH714_034861	0.025	0.225	0.111	Segmental duplication	Purifying selection
*HbCAT1*	GH714_028220	*HbCAT4*	GH714_007303	0.096	1.660	0.058	Segmental duplication	Purifying selection
*HbCAT2*	GH714_016778	*HbCAT4*	GH714_007303	0.038	0.351	0.107	Segmental duplication	Purifying selection
*HbCAT2*	GH714_016778	*HbCAT3*	GH714_034861	0.086	1.472	0.058	Segmental duplication	Purifying selection
*HbCAT2*	GH714_016778	*HbCAT5*	GH714_007170	0.036	0.273	0.131	Segmental duplication	Purifying selection
*HbCAT3*	GH714_034861	*HbCAT4*	GH714_007303	0.101	1.417	0.071	Segmental duplication	Purifying selection
*HbCAT4*	GH714_007303	*HbCAT5*	GH714_007170	0.020	0.108	0.187	Segmental duplication	Purifying selection

Note: Groups A and B represent collinear gene pairs of *HbCAT* genes in *H. brasiliensis*.

## Data Availability

The data presented in this study are available in the Supplementary Materials.

## Supplementary Materials

The following supporting information can be downloaded at: https://www.mdpi.com/article/10.3390/ijms25010070/s1.
